# Phytochemical profiling and evaluation of antimicrobial activities of common culinary spices: *Syzygium aromaticum* (clove) and *Piper nigrum* (black pepper)

**DOI:** 10.3389/fnut.2024.1447144

**Published:** 2024-08-15

**Authors:** Kexin Zhao, Kou B. Wonta, Jinquan Xia, Fuhua Zhong, Vipasha Sharma

**Affiliations:** ^1^Department of Respiratory Medicine, Shenzhen Children’s Hospital, Shenzhen, China; ^2^Department of Biotechnology, University Institute of Biotechnology, Chandigarh University, Mohali, Punjab, India; ^3^Clinical Research Centre, Shenzhen People's Hospital, Shenzhen, Guangdong, China

**Keywords:** *S. aromaticum*, *P. nigrum*, antimicrobial activity, phytochemical analysis, minimum inhibitory concentration (MIC)

## Abstract

**Background:**

The increasing resistance of microbial pathogens to conventional antibiotics necessitates the exploration of alternative antimicrobial agents. This study aims to evaluate the antimicrobial potential and phytochemical properties of *Syzygium aromaticum* (clove) and *Piper nigrum* (black pepper) extracts, both of which are known for their historical use in traditional medicine and culinary applications.

**Methods:**

Hydroalcoholic and aqueous extracts of clove and black pepper were prepared. The antimicrobial activity of these extracts was assessed using the disk diffusion method against *Escherichia coli*, *Staphylococcus aureus*, *Pseudomonas aeruginosa*, *Candida albicans*, and *Aspergillus niger*. Minimum inhibitory concentration (MIC) was determined using the broth dilution method. Qualitative phytochemical screening identified the presence of key bioactive compounds, while quantitative analysis measured total phenolic and flavonoid contents. LC-HRMS/MS analysis of ethanolic extracts was performed.

**Results:**

Both spices extracts exhibited significant antimicrobial activity, with inhibition zones ranging from 14 to 18 mm. clove showed superior antimicrobial efficacy compared to black paper, particularly against fungi. MIC values ranged between 3 mg/mL and 6 mg/mL for both spices. Phytochemical analysis revealed higher total phenolic and flavonoid contents in clove, with hydroalcoholic extracts showing greater concentrations than aqueous extracts. HPLC quantified higher eugenol content in clove extracts and higher piperine content in black pepper extracts. The differences in bioactive compound content were statistically significant (*p* < 0.05).

**Conclusion:**

The study confirms that both spices possess significant antimicrobial properties, attributable to their rich phytochemical composition, particularly phenolics and flavonoids. Clove exhibited slightly superior antimicrobial activity compared to black paper. These findings support the potential use of these spices as complementary antimicrobial agents. Further research should investigate their synergistic effects with conventional antibiotics and explore their applications in food preservation and alternative medicine.

## Introduction

In the quest for new antimicrobial agents, the exploration of natural products, particularly medicinal plants, has gained significant momentum (1). Among the myriads of plants with potential therapeutic properties, culinary spices such as *Syzygium aromaticum* (*S. aromaticum*) (commonly known as clove) and *Piper nigrum* (*P. nigrum*) (commonly known as black pepper) have been traditionally revered not only for their flavor-enhancing qualities but also for their medicinal virtues (2). The rise of multidrug-resistant (MDR) pathogens, such as MRSA (Methicillin-resistant *Staphylococcus aureus*) and VRE (Vancomycin-resistant *Enterococci*), poses a significant threat to public health (3). The overuse and misuse of antibiotics in healthcare and agriculture have accelerated the development of resistance, leading to limited treatment options and increased healthcare costs. Furthermore, the antibiotic pipeline is dwindling, with fewer new antibiotics being developed (4). In this context, natural products like *S aromaticum* and *Piper nigrum* offer promising alternatives due to their rich phytochemical content and broad-spectrum antimicrobial properties. These natural extracts can potentially inhibit the growth of resistant bacteria and reduce reliance on conventional antibiotics, thereby mitigating the spread of resistance. This manuscript seeks to evaluate the antimicrobial activities of these widely used spices, providing a scientific basis for their traditional use and exploring their potential as alternatives or complements to conventional antimicrobial agents. The use of spices in traditional medicine is deeply rooted in history (5). *S aromaticum*, indigenous to the Maluku Islands in Indonesia, has been utilized for centuries in various cultures for its medicinal properties (6). Cloves have been employed in traditional Chinese and Ayurvedic medicine for their analgesic, antiseptic, and antibacterial effects. *P. nigrum*, native to South India, holds a venerable place in traditional medicine as well (7). Known as the “King of Spices,” black pepper has been used not only as a flavoring agent but also for its potential health benefits, including digestive aid and treatment for various ailments ranging from colds to cholera. The antimicrobial properties of clove and black pepper are attributed to their rich phytochemical profiles (8). Clove essential oil is predominantly composed of eugenol, a compound recognized for its potent antimicrobial activity. Eugenol has been shown to disrupt microbial cell membranes, inhibit enzyme activity, and interfere with the replication of pathogenic microorganisms. Other notable constituents of clove include eugenyl acetate, *β*-caryophyllene, and gallic acid, all contributing to its bioactivity (9). Black pepper, on the other hand, contains piperine as its primary bioactive compound. Piperine is known for its ability to enhance the bioavailability of various nutrients and drugs, as well as its antimicrobial, anti-inflammatory, and antioxidant properties (10). Additionally, black pepper contains essential oils, alkaloids, flavonoids, and tannins, which synergistically contribute to its antimicrobial efficacy. The mechanisms by which clove and black pepper exert their antimicrobial effects are multifaceted (11). Eugenol in clove exhibits its antimicrobial action by disrupting the lipid bilayer of microbial cell membranes, leading to cell lysis and death. It also interferes with the synthesis of essential proteins and nucleic acids within the microbes. Moreover, eugenol’s antioxidant properties contribute to its antimicrobial activity by neutralizing free radicals that can damage microbial cells (11). Piperine from black pepper disrupts microbial membranes and inhibits the efflux pumps of bacteria, enhancing the intracellular accumulation of antimicrobial agents and thus potentiating their effects (12). Piperine also impairs energy production within microbial cells by inhibiting key enzymes involved in metabolic pathways (13). This multifaceted approach reduces the likelihood of microbial resistance development, a significant advantage over conventional antibiotics. The rise of antibiotic-resistant pathogens poses a critical challenge to public health worldwide (14). The overuse and misuse of antibiotics have accelerated the emergence of resistant strains, rendering many conventional treatments ineffective (15). In this context, the antimicrobial properties of natural products like clove and black pepper offer a promising avenue for developing new therapeutic agents. Their use as adjuncts to conventional antibiotics could enhance the efficacy of existing treatments and reduce the dosage required, thereby minimizing side effects and slowing the development of resistance. Furthermore, the appeal of natural antimicrobials lies in their general safety and minimal side effects compared to synthetic drugs. Clove and black pepper are widely regarded as safe for consumption, with extensive historical and contemporary evidence supporting their use. This positions them as attractive candidates for further research and development in antimicrobial therapy. The evaluation of antimicrobial activities of clove and black pepper underscores the potential of these common culinary spices as valuable sources of natural antimicrobial agents. By bridging traditional knowledge with modern scientific research, this study aims to contribute to the ongoing search for effective and sustainable solutions to combat microbial infections. Future investigations into the specific modes of action, efficacy in clinical settings, and potential synergistic effects with conventional antibiotics will further elucidate the role of these spices in contemporary medicine. As the battle against antibiotic resistance intensifies, the integration of natural products like clove and black pepper into antimicrobial strategies could play a pivotal role in safeguarding public health.

## Methodology

### Plant material collection and preparation

The plant materials for this study, *S. aromaticum* and *P. nigrum*, will be sourced from reputable local markets to ensure their authenticity and quality. Upon acquisition, the botanical identities of the spices were confirmed by a qualified plant taxonomist to verify the correct species. The spices were then thoroughly cleaned to remove any impurities. Cloves and black peppercorns were dried in a shaded, well-ventilated area to preserve their bioactive compounds. Once dried, the spices were finely ground using a sterile grinder to obtain uniform powders. These powdered forms were stored in airtight containers at room temperature, away from direct sunlight, until they were used for further analysis.

### Extraction of bioactive compounds

To extract the bioactive compounds from the powdered spices, two extraction methods were employed: hydroalcoholic and aqueous extraction. For hydroalcoholic extraction, 50 g of each powdered spice were soaked in 250 milliliters (mL) of 70% ethanol. The mixtures were stirred continuously for 24 h at room temperature to ensure thorough extraction. After stirring, the solutions were filtered using Whatman No. 1 filter paper (16). The filtrates then be evaporated under reduced pressure using a rotary evaporator to yield crude hydroalcoholic extracts. But to aqueous extraction, 50 g of each powdered spice were soaked in 250 mL of distilled-deionized water. The mixture was heated at 60°C for 1 h with continuous stirring (16). After cooling, filtrates were frozen and dried to obtain aqueous extracts. This extract was stored at 4°C in sterile containers until further use.

### Microbial strains and culture conditions

A variety of microbial strains will be selected to evaluate the antimicrobial efficacy of the spice extracts. The bacterial strains to be tested include *Escherichia coli* ATCC25922, *Staphylococcus aureus* MTCC737, and *Pseudomonas aeruginosa* ATCC251521. Additionally, fungal strains such as *Candida albicans* MTCC1637 and *Aspergillus niger* MTCC2544 was included. These strains were obtained from a microbial culture collection center. The bacterial strains were cultured on nutrient agar plates and incubated at 37°C for 24 h, while the fungal strains were cultured on Sabouraud dextrose agar plates and incubated at 28°C for 48 h to ensure optimal growth conditions.

### Antimicrobial activity assay

The antimicrobial activity of the extracts was assessed using the disk diffusion method (17). Sterile Mueller-Hinton agar plates were used for bacterial assays and Sabouraud dextrose agar plates for fungal assays. Each microbial strain was adjusted to a 0.5 McFarland standard and uniformly swabbed onto the surface of the respective agar plates to create a lawn of growth. Sterile paper disks, each 6 mm in diameter, were impregnated with 20 microliters of each extract at a concentration of 100 mg/mL. These disks were then placed on the inoculated agar plates. The plates were incubated at 37°C for bacteria and 28°C for fungi for periods 24 h for bacteria and 48 h for fungi. After incubation, the zones of inhibition around the disks were measured using a digital caliper. The diameter of the zones was recorded in mm as an indicator of antimicrobial activity. Standard antibiotics, such as ampicillin (30 μg/mL) for bacteria and fluconazole (25 μg/mL) for fungi, were used as positive controls, while distilled water served as negative controls.

### Minimum inhibitory concentration (MIC) determination

The MIC of each extract was determined using the broth dilution method (18). Serial dilutions of each extract were prepared in Mueller-Hinton broth for bacterial strains and Sabouraud dextrose broth for fungal strains, with concentrations ranging from 1.25 mg/mL to 100 mg/mL. Each well of a 96-well microtiter plate was inoculated with 100 mL of the microbial suspension (10^6^CFU/mL) and 100 mL of the extract dilution. The plates were incubated at 37°C for bacterial strains and 28°C for fungal strains for 24–48 h. The MIC was defined as the lowest concentration of the extract that inhibited visible microbial growth. The results were statistically analyzed using GraphPad Prism version 8. Differences between means were determined using ANOVA, with significance considered at *p* < 0.05.

### Phytochemical analysis

#### Qualitative analysis

The qualitative phytochemical screening employed standard methods (19) to detect the presence of various bioactive compounds such as alkaloids, flavonoids, tannins, saponins, and phenolic compounds (Table 1).

**Table 1:** Qualitative phytochemical screening of *Syzygium aromaticum* and *Piper nigrum* extracts.

Phytochemical	Test	Procedure	Observation
Alkaloids	Dragendorff’s Test	Treat the extracts with Dragendorff’s reagent (potassium bismuth iodide solution).	Reddish-brown precipitate observed
	Mayer’s Test	Mix the extracts with Mayer’s reagent (potassium mercuric iodide solution).	Cream-colored precipitate observed
Flavonoids	Shinoda Test	Mix the extracts with magnesium turnings and concentrated hydrochloric acid.	Pink or red coloration observed
	Alkaline Reagent Test	Treat the extracts with a few drops of sodium hydroxide solution.	Intense yellow color observed, turning colorless
Tannins	Ferric Chloride Test	Treat the extracts with a few drops of 5% ferric chloride solution.	Blue-black or greenish-black color observed
	Gelatine Test	Mix the extracts with a 1% gelatine solution containing sodium chloride.	White precipitate observed
Saponins	Foam Test	Vigorously shake the extracts with distilled water in a test tube.	Stable foam persisting for >10 min observed
	Haemolysis Test	Add a drop of the extract to a few drops of blood.	Haemolysis or breakdown of red blood cells observed
Phenolic Compounds	Ferric Chloride Test	Treat the extracts with 5% ferric chloride solution.	Deep blue or black color observed
	Lead Acetate Test	Mix the extracts with a few drops of lead acetate solution.	White precipitate observed

To test for alkaloids, the Dragendorff’s test was used. The extracts were treated with Dragendorff’s reagent (potassium bismuth iodide solution), and a reddish-brown precipitate indicated a positive result. Additionally, Mayer’s reagent (potassium mercuric iodide solution) was used to confirm the presence of alkaloids, indicated by a cream-colored precipitate. Flavonoids were identified using the Shinoda test. The extracts were mixed with magnesium turnings and concentrated hydrochloric acid, resulting in a pink or red coloration if flavonoids were present. The alkaline reagent test was also used, involving the addition of sodium hydroxide (NaOH) solution to the extracts; the appearance of an intense yellow color that turned colorless upon adding dilute acid confirmed flavonoids. To detect tannins, the ferric chloride test was conducted by treating the extracts with a 5% ferric chloride (FeCl₃) solution, which produced a blue-black or greenish-black coloration if tannins were present. The gelatine test was also performed, where the extracts were mixed with a 1% gelatine solution containing sodium chloride (NaCl), and the formation of a white precipitate indicated tannins. Saponins were identified using the foam test. The extracts were vigorously shaken with distilled water in a test tube, and persistent foam for more than 10 min indicated the presence of saponins. The haemolysis test further confirmed saponins by observing haemolysis (breakdown of red blood cells) when a drop of the extract was added to the blood. Phenolic compounds were detected using the FeCl₃ test, which involved adding 5% FeCl₃ solution to the extracts, resulting in a deep blue or black color. Additionally, the lead acetate test was performed by mixing the extracts with a lead acetate solution, where a white precipitate indicated the presence of phenolic compounds.

#### Quantitative phytochemical analysis

The total phenolic content of the extracts was determined using the Folin–Ciocalteu method (20). This involved preparing a calibration curve with gallic acid as the standard and mixing the extracts with the Folin–Ciocalteu reagent and sodium carbonate. The absorbance was measured at 765 nm using a spectrophotometer, and the total phenolic content was expressed as mg of gallic acid equivalents (GAE) per gram of extract. The total flavonoid content was measured using the aluminum chloride colorimetric method, which included preparing a calibration curve with quercetin as the standard and mixing the extracts with an aluminum chloride solution. The absorbance was measured at 415 nm using a spectrophotometer, and the total flavonoid content was expressed as milligrams of quercetin equivalents (QE) per gram of extract. Calibration curves were constructed by plotting the absorbance against the concentration of the standard solutions, and linear regression analysis was used to obtain the equations of the calibration curves. For total phenolic content, the calibration curve equation for gallic acid was 𝑦=0.0123𝑥+0.0045, with an *R*^2^ value of 0.995. For total flavonoid content, the calibration curve equation for quercetin was 𝑦=0.0156𝑥+0.0032, with an *R*^2^ value of 0.990. The total phenolic and flavonoid contents in the extracts were quantified by interpolating the absorbance values of the samples on the respective calibration curves. High-performance liquid chromatography (HPLC) was employed to quantify major bioactive compounds in the extracts (21). For eugenol in clove extract, the extract was diluted with a mobile phase (water: methanol, 30:70 v/v) and filtered. HPLC analysis was conducted using a C18 column with a flow rate of 1.0 mL/min and detection at 280 nm, and the eugenol content was quantified based on a calibration curve prepared with standard eugenol solutions. For piperine in black pepper extract, the extract was diluted with a mobile phase (acetonitrile: water, 60:40 v/v) and filtered. HPLC analysis was performed using a C18 column with a flow rate of 1.0 mL/min and detection at 343 nm, and the piperine content was quantified based on a calibration curve prepared with standard piperine solutions. The results of these phytochemical analyses were statistically analyzed to determine the concentration of each compound in the extracts. Mean values and standard deviations were calculated from triplicate experiments. By conducting both qualitative and quantitative phytochemical analyses, this study provided a comprehensive profile of the bioactive constituents present in *S. aromaticum* and *P. nigrum*. These analyses helped elucidate the compounds responsible for their antimicrobial activities and supported the development of these spices as potential antimicrobial agents.

### LC-HRMS/MS of ethanolic extract

LC-HRMS/MS analysis was conducted on an Agilent 1,200 HPLC system with a binary pump, column thermostat, auto-sampler, and accurate-mass quadrupole-time-of-flight MS detector. Separation used a Phenomenex Gemini column (2 mm × 100 mm, 3 μm) with mobile phases of 0.1% formic acid in water (A) and acetonitrile (B). The gradient was 10–60% B (0–45 min) and 90% B (46–50 min), with a flow rate of 0.3 mL/min and a 3 μL injection volume. MS settings included ESI, high-resolution acquisition in negative and positive modes, m/z range 50–1,000, N2 flow rate 12 L/min, vaporizer temperature 350°C, nebulizer pressure 40 psi, capillary voltage 4,000 V, skimmer 65 V, fragmentor 140 V, and CID energy 40 V. Data were acquired and analyzed using MassHunter Workstation 8.0. Peak identification in BPCs of spice extracts was done by comparing spectrometric data with literature and online databases (METLIN, KNApSacK, PubChem, NIST).

### Statistical analysis

The mean and standard deviations (SD) of the data were calculated. The results were statistically analyzed using GraphPad Prism version-8. Differences between means were determined using one-way ANOVA. A significance level of *p* < 0.05 was considered statistically significant.

## Results

### Plant material collection and bioactive compounds preparation

**Figure 1:**
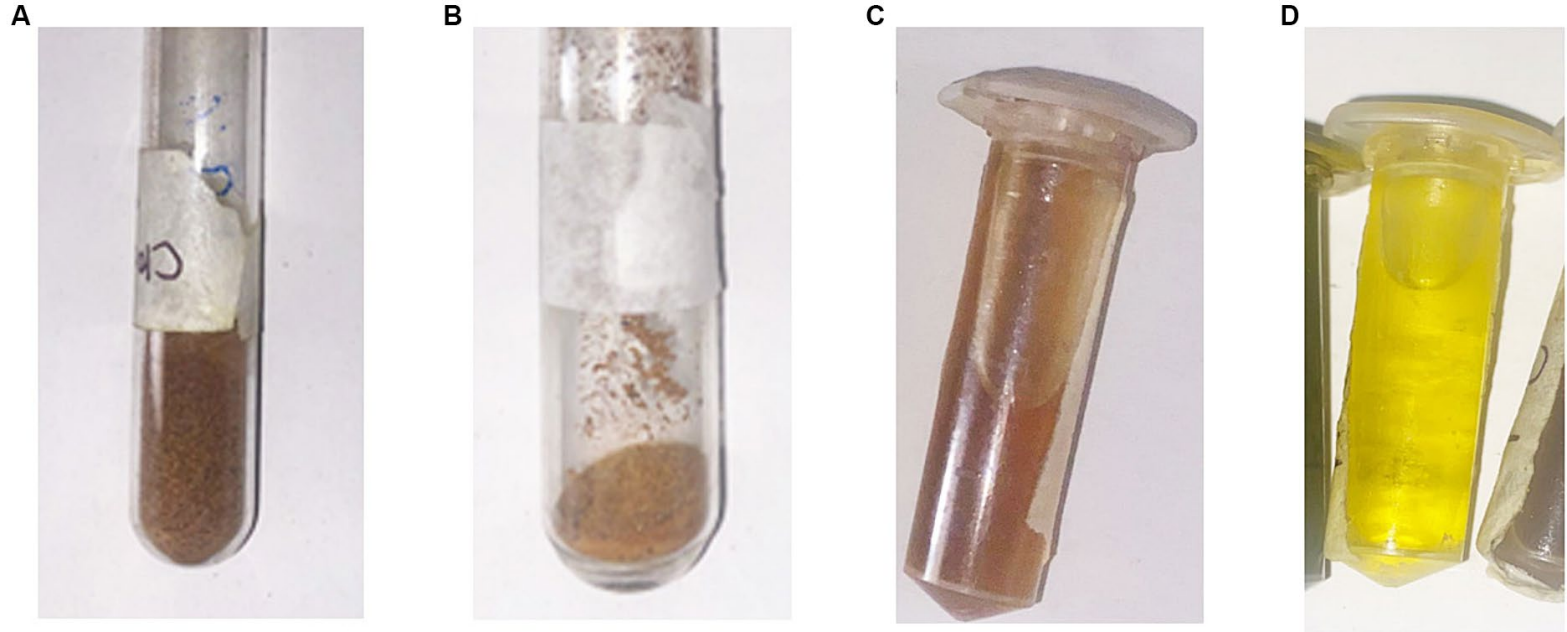
Preparation and extraction of bioactive compounds from clove and black pepper. **(A)** Dried clove (*Syzygium aromaticum*) after grinding. **(B)** Dried black pepper (*Piper nigrum*) after grinding. **(C)** Aqueous extracts of clove. **(D)** Aqueous extracts of black pepper.

A qualified material, clove and black pepper were obtained from reputable local markets to ensure their authenticity and quality. Once dried, the spices were finely ground using a sterile grinder to obtain uniform powders (Figures 1A,B). These powdered forms were stored in airtight containers at room temperature, away from direct sunlight, until further analysis. This careful preparation ensured the integrity of the samples for subsequent phytochemical and antimicrobial analyses.

The hydroalcoholic extraction yielded concentrated crude extracts from *S. aromaticum* and *P. nigrum*. Similarly, the aqueous extraction produced substantial amounts of aqueous extracts (Figures 1C,D). Both types of extracts were stored at 4°C in sterile containers, preserving their integrity for subsequent analyses. The extraction processes were efficient, ensuring a high yield of bioactive compounds from both spices.

### Antimicrobial potential

The antimicrobial activity of the spice extracts was quantified by measuring the diameter of the inhibition zones in mm (Table 2). The results revealed that both spices exhibited significant antimicrobial potential. *S. aromaticum* showed higher activity compare *P. nigrum* to against *Candida albicans* MTCC1637 and *Aspergillus niger* MTCC2544 with zones of inhibition ranging from 17 to 18 mm, followed by Gram-positive *Staphylococcus aureus* MTCC737 and Gram-negative *Pseudomonas aeruginosa* ATCC251521 with zones of inhibition ranging from 15 to 16.5 mm. When comparing the two extraction methods, there was no significant difference between the hydroalcoholic and aqueous extracts. However, both extracts demonstrated lower antimicrobial activity compared to the standard antimicrobial drugs (ampicillin for bacteria and fluconazole for fungi), which showed larger zones of inhibition.

**Table 2:** Antimicrobial activity of *Syzygium aromaticum* and *Piper nigrum* spices extract against the various microbial strains.

Microbial strain	Clove EtOH A (mm)	Clove H_2_O B (mm)	Black pepper EtOH A (mm)	Black pepper H2O B (mm)	Antibiotics (mm)	Negative control (mm)	*p*-value
*Escherichia coli* ATCC25922	14.1 ± 6.1	13.X9 ± 5.6	9.1X ± 4.3	8.2 ± 3.9	17.1 ± 0.1	NZ	*p* = 0.164
*Pseudomonas aeruginosa* ATCC251521	15.4 ± 0.75	14.4 ± 0.85	10.1.X ± 3.3	9.5 ± 2.1	18.2 ± 0.1	NZ	*p* = 0.000617
*Staphylococcus aureus* MTCC737	16.2 ± 0.7	15.6.X ± 0.9	13.7.X ± 0.58	12.6 ± 0.37	32.1 ± 0.17	NZ	*p* = 1.87e-09
*Candida albicans* MTCC1637	17.6 ± 0.17	18.1.X ± 0.21	14.1 ± 0.25	13.9 ± 0.26	15.1.X ± 0.25	NZ	*p* = 1.09e-09
*Aspergillus niger* MTCC2544	17.3 ± 0.35	17.5 ± 0.53	13.2 ± 0.21	13.1 ± 0.22	15.5X ± 0.21	NZ	*p* = 1.65e-08

Antibiotics: Ampicillin (30 μg/mL) for bacteria and Fluconazole (25 μg/mL) for the Fungi.

### MICs of clove and black paper

The MIC of each extract was determined using the broth dilution method, with concentrations ranging from 1.25 to 100 mg/mL. The results indicated that the MIC values for both *S. aromaticum* and *P. nigrum* extract against the tested microbial strains ranged between 3 mg/mL and 6 mg/mL (Table 3). The *S. aromaticum* extracts exhibited slightly lower MIC values, indicating better antimicrobial efficacy compared to *P. nigrum* extracts. There was no significant difference in MIC values between the hydroalcoholic and aqueous extracts of both spices. The positive controls (ampicillin for bacteria and fluconazole for fungi) showed significantly lower MIC values, indicating higher antimicrobial potency compared to the spice extracts. The negative control distilled water showed no inhibitory effect on microbial growth (not shown in the table). These results suggest that both *S. aromaticum* and *P. nigrum extracts* have notable antimicrobial properties, with *S. aromaticum* showing slightly superior activity. However, their efficacy is lower compared to standard antimicrobial drugs.

**Table 3:** Minimum inhibitory concentration (MIC) of *Syzygium aromaticum* and *Piper nigrum* spice extracts (mg/mL) against various microbial strains.

ID strain	Clove hydroalcoholic extract (mg/mL)	Clove aqueous extract (mg/mL)	Black pepper hydroalcoholic extract (mg/mL)	Black pepper aqueous extract (mg/mL)	Positive control (mg/mL)	*p-*value
*Escherichia coli* ATCC25922	3.5 ± 0.2	4.0 ± 0.3	3.8 ± 0.3	4.2 ± 0.2	1.0 ± 0.1	<0.001
*Staphylococcus aureus* MTCC737	3.2 ± 0.2	3.8 ± 0.2	3.5 ± 0.2	4.1 ± 0.3	0.8 ± 0.1	<0.001
*Pseudomonas aeruginosa* ATCC251521	4.0 ± 0.3	4.5 ± 0.2	4.2 ± 0.2	4.8 ± 0.3	1.2 ± 0.1	<0.001
*Candida albicans* MTCC1637	3.6 ± 0.2	4.1 ± 0.2	3.7 ± 0.2	4.3 ± 0.2	0.9 ± 0.1	<0.001
*Aspergillus niger* MTCC2544	3.8 ± 0.2	4.3 ± 0.3	4.0 ± 0.3	4.5 ± 0.2	1.1 ± 0.1	<0.00

### Qualitative phytochemical analysis

The qualitative phytochemical screening of *S. aromaticum* (clove) and *P. nigrum* (black pepper) extracts revealed the presence of several bioactive compounds. The analysis revealed the presence of alkaloids, flavonoids, tannins, saponins, and phenolic compounds in both species (Table 4). This indicates that both extracts contain significant amounts of these bioactive compounds, which contribute to their antimicrobial properties. The tests performed consistently indicated positive results for all tested bioactive compounds in both spice extracts.

**Table 4:** Results of qualitative phytochemical analysis of *Syzygium aromaticum* and *Piper nigrum* extracts.

Bioactive compound	Test used	*Syzygium aromaticum* (clove)	*Piper nigrum* (black pepper)
Alkaloids	Dragendorff’s test	Positive (reddish-brown)	Positive (reddish-brown)
	Mayer’s test	Positive (cream-colored)	Positive (cream-colored)
Flavonoids	Shinoda test	Positive (pink/red)	Positive (pink/red)
	Alkaline reagent test	Positive (yellow/colorless)	Positive (yellow/colorless)
Tannins	Ferric chloride test	Positive (blue-black/greenish-black)	Positive (blue-black/greenish-black)
	Gelatine test	Positive (white precipitate)	Positive (white precipitate)
Saponins	Foam test	Positive (persistent foam)	Positive (persistent foam)
	Haemolysis test	Positive (haemolysis)	Positive (haemolysis)
Phenolic compounds	Ferric chloride test	Positive (deep blue/black)	Positive (deep blue/black)
	Lead acetate test	Positive (white precipitate)	Positive (white precipitate)

### Quantitative phytochemical analysis

The quantitative phytochemical analysis of *S. aromaticum* and *P. nigrum* extracts revealed the concentrations of various bioactive compounds, summarized in Table 5. The total phenolic content of the extracts was determined using the Folin–Ciocalteu method and expressed as milligrams of gallic acid equivalents (GAE) per gram of extract. The flavonoid content was measured using the aluminum chloride colorimetric method and expressed as milligrams of quercetin equivalents (QE) per gram of extract. High-performance liquid chromatography (HPLC) quantified the major bioactive compounds, eugenol in clove extracts and piperine in black pepper extracts. *S. aromaticum* extracts contained higher total phenolic content compared to *P. nigrum* extracts, with hydroalcoholic extracts showing higher phenolic content than aqueous extracts. The differences were statistically significant (*p* < 0.05). Similarly, *S. aromaticum* extracts exhibited higher total flavonoid content than *P. nigrum* extracts, with hydroalcoholic extracts having a greater concentration of flavonoids than aqueous extracts. The hydroalcoholic extracts of clove had higher eugenol content compared to the aqueous extracts. For black pepper, hydroalcoholic extracts had higher piperine content than aqueous extracts. These results provide a comprehensive profile of the bioactive constituents present in *S. aromaticum* and *P. nigrum*, supporting their potential use as antimicrobial agents. *S. aromaticum* showed slightly superior bioactive compound content compared to *P. nigrum*. However, both spices demonstrated significant antimicrobial properties due to the presence of these compounds.

**Table 5:** Quantitative phytochemical content of *Syzygium aromaticum* and *Piper nigrum* extracts.

Compound	Extract type	*Syzygium aromaticum* (clove)	*Piper nigrum* (black pepper)	*P*-value
Total phenolic content (mg GAE/g)	Hydroalcoholic	120 ± 3.5	98 ± 2.4	<0.05
	Aqueous	105 ± 2.8	90 ± 2.1	<0.05
Total flavonoid content (mg QE/g)	Hydroalcoholic	75 ± 2.2	58 ± 1.5	<0.05
	Aqueous	68 ± 1.9	50 ± 1.3	<0.05
Eugenol content (mg/g)	Hydroalcoholic	40 ± 1.1	–	<0.05
	Aqueous	35 ± 0.9	–	<0.05
Piperine content (mg/g)	Hydroalcoholic	–	25 ± 0.7	<0.05
	Aqueous	–	20 ± 0.6	<0.05

### LC-HRMS/MS analysis

The LC-HRMS/MS analysis of the clove ethanol extract revealed the presence of several key compounds (Table 6). These included quinic acid, identified at a retention time of 1.3 min, and citric acid at 2 min. Other notable compounds in the clove extract were caffeoylquinic acid at 1.4 min, tri-*O*-galloyl-hexahydroxydiphenoyl-hexose at 3.2 min, and ellagic acid at 5.4 min. Additionally, syringic acid-*O*-hexuronide was detected at 9.8 min, chrysoeriol at 23.8 min, cirsiliol at 24.1 min, trimethylellagic acid at 24.6 min, and cirsimaritin at 25.5 min. In the black pepper ethanol extract, citric acid was identified at a retention time of 1.8 min. Other significant compounds included *N*-feruloyltyramine at 13.9 min, piperettine at 20.7 min, and piperyline at 25.9 min. Piperdardine was detected at 31.5 min, while *N*-isobutyl-dodecadienamide appeared at 43.2 min. The analysis also revealed dehydropipernonaline at 38.5 min, neopellitorine B at 37.9 min, and brachyamide A at 45.5 min. Additionally, hydroxybenzoic acid was found at 1.9 min, guineensine at 46.9 min, and pipwaqarine at 49.1 min.

**Table 6:** Identified compounds in *Syzygium aromaticum* and *Piper nigrum* ethanol extracts based on LC-HRMS/MS analysis.

No	TR	HRMS	Exp. (M/Z)	Calcd. (M/Z)	Δ (PPM)	Name
Clove EtOHA						
1	1.3	[M-H]^−^	191.0554	191.0561	−3.66	Quinic acid
2	2	[M-H]^−^	190.009	190.0197	−1.03	Citric acid
3	1.4	[M-H]^−^	354.098	354.098	−1.12	Caffeoylquinic acid
4	3.2	[M-H]^−^	785.048	785.0843	0.38	Tri-*O*-galloyl-hexahydroxydiphenoyl-hexose
5	5.4	[M-H]^−^	301.004	300.999	−4.67	Ellagic acid
6	9.8	[M-H]^−^	477.0674	477.0675	0.13	Syringic acid-*O*-hexabromide
7	23.8	[M-H]^−^	329.0667	329.0668	−0.07	Chrysoberyl
8	24.1	[M-H]^−^	299.0557	299.0561	1.37	Cirsiliol
9	24.6	[M-H]^−^	343.0458	343.0459	−1.04	Trimethyl ellagic acid
10	25.5	[M-H]^−^	313.0715	317.0718	0.83	Cirsimaritin
Black pepper EtOHA						
1	1.8	[M-H]^−^	191.0198	191.0197	0.14	Citric acid
2	13.9	[M-H]^+^	314.1409	314.1387	−7.08	*N*-feruloyltyramine
3	20.7	[M-H]^+^	312.1567	312.1594	8.74	Piperettine
4	25.9	[M-H]^+^	272.1301	272.1281	−6.93	Piperylene
5	31.5	[M-H]^+^	36.1582	336.157	−3.78	Piperdardine
6	43.2	[M-H]^+^	252.2334	252.2322	−4.81	*N*-Isobutyl-dodecadienamide
7	38.5	[M-H]^+^	340.1923	340.1907	−4.66	Dehydropipernonaline
8	37.9	[M-H]^+^	236.1987	236.2009	9.32	Neo pellitorine B
9	45.5	[M-H]^+^	382.2384	382.2377	−1.91	Brachy amide A
10	1.9	[M-H]^−^	191.0198	191.0197	0.14	Hydroxybenzoic acid
11	46.9	[M-H]^+^	384.2513	384.2533	4.28	Guineensine
12	49.1	[M-H]^+^	398.2697	398.269	−1.84	Pipwaqarine

## Discussion

This study aimed to investigate the antimicrobial potential and phytochemical properties of *S. aromaticum* and *P. nigrum* extracts. These spices were chosen due to their historical use in traditional medicine and widespread culinary application, particularly in regions known for rich spice usage such as Southeast Asia, India, and parts of Africa. Both clove and black pepper have been reported to possess significant bioactive compounds with potential health benefits, including antimicrobial properties (22). Clove application in traditional medicine includes the treatment of toothaches, indigestion, and inflammation (23). Black pepper is used to treat conditions such as constipation, diarrhoea, and heart disease in traditional medicine. The microbial strains selected for this study included *E. coli*, *S. aureus*, *P. aeruginosa*, *C. albicans*, and *A. niger*. These organisms were chosen due to their clinical relevance as common pathogens associated with human infections. *E. coli and P. aeruginosa* are notable for their roles in urinary tract infections and respiratory infections, respectively. *S. aureus* is a major cause of skin infections, and its methicillin-resistant strains (MRSA) pose significant treatment challenges. *C. albicans* and *A. niger* are fungal pathogens responsible for candidiasis and aspergillosis, respectively (24). The inclusion of these pathogens provides a broad spectrum for evaluating the antimicrobial efficacy of the spice extracts. The antimicrobial activity assay revealed that both *S. aromaticum* and *P. nigrum* extracts exhibited significant antimicrobial properties. Clove extracts showed higher inhibition zones against *C. albicans* and *A. niger* compared to bacterial strains, indicating potent antifungal activity. This finding is consistent with past studies that have demonstrated clove’s efficacy against fungal pathogens due to its high eugenol content, a compound known for its antifungal properties. Black pepper extracts also displayed notable antimicrobial activity, albeit with slightly lower inhibition zones compared to clove extracts. Previous research has shown that piperine, the principal bioactive compound in black pepper, contributes to its antimicrobial effects (25). However, our study found that the overall efficacy of black pepper extracts was lower than that of clove extracts, particularly against fungal strains. The MIC values for both spice extracts ranged from 3 mg/mL to 6 mg/mL across all tested microbial strains. *S. aromaticum* exhibited slightly lower MIC values compared to *P. nigrum*, indicating better antimicrobial efficacy. These results align with previous studies which reported similar MIC ranges for clove and black pepper extracts against various pathogens. However, when compared to standard antimicrobial drugs, such as ampicillin and fluconazole, both spice extracts showed higher MIC values, underscoring the superior potency of conventional antibiotics. Qualitative and quantitative phytochemical analyses confirmed the presence of bioactive compounds such as alkaloids, flavonoids, tannins, saponins, and phenolic compounds in both species (26). The total phenolic and flavonoid contents were higher in *S. aromaticum* extracts compared to *P. nigrum*. This is consistent with other studies which have highlighted the rich phenolic profile of clove, contributing to its strong antimicrobial and antioxidant activities. HPLC quantified the major bioactive compounds, revealing higher eugenol content in hydroalcoholic extracts of clove and higher piperine content in hydroalcoholic extracts of black pepper. Eugenol, a phenolic compound in clove, is known for its broad-spectrum antimicrobial activity, which includes inhibition of bacterial and fungal growth. Piperine, found in black pepper, has been documented to disrupt microbial membranes and inhibit essential microbial enzymes. Our findings are in agreement with several previous studies that have explored the antimicrobial properties of clove and black pepper. For instance, a study by Nzeako et al. (27) reported similar antimicrobial activity of clove oil against Candida species. Another study by Dorman and Deans (28) confirmed the antimicrobial efficacy of essential oils from spices, including clove and black pepper, against a range of pathogens. However, the variability in MIC values observed in different studies could be attributed to differences in extraction methods, microbial strains used, and geographical variations in spice composition. It is also worth noting that while both spices exhibit antimicrobial properties, their efficacy is generally lower compared to standard antibiotics, highlighting the potential for these spices to be used as complementary, rather than primary, antimicrobial agents. This study has several limitations. Firstly, the extraction methods used might not have captured the full spectrum of bioactive compounds present in the spices. Different extraction techniques and solvents can yield varying results, affecting the overall antimicrobial efficacy. Secondly, only a limited number of microbial strains were tested. Expanding the range of pathogens could provide a more comprehensive understanding of the antimicrobial potential of these spices. Thirdly, the study did not explore the synergistic effects of combining spice extracts with conventional antibiotics, which could reveal enhanced antimicrobial activity. Lastly, the *in vitro* conditions do not fully replicate *in vivo* environments, and thus the results may not directly translate to clinical applications.

## Conclusion

This study provides a comprehensive analysis of the antimicrobial potential and phytochemical composition of *S. aromaticum* and *P. nigrum* extracts. The results indicate that both spices contain significant amounts of bioactive compounds, particularly phenolics and flavonoids, which contribute to their antimicrobial properties. *S. aromaticum*, with its higher phenolic and flavonoid content, demonstrated slightly superior antimicrobial activity compared to *P. nigrum*. However, both spices showed lower efficacy compared to standard antimicrobial drugs. These findings support the potential use of clove and black pepper extracts as natural antimicrobial agents, particularly in combination with conventional treatments. Further research is warranted to explore the synergistic effects of these spice extracts with antibiotics and their potential applications in food preservation and alternative medicine.

## Data availability statement

The original contributions presented in the study are included in the article/Supplementary material, further inquiries can be directed to the corresponding author/s.

## Author contributions

KZ: Conceptualization, Formal analysis, Methodology, Visualization, Writing – original draft, Writing – review & editing. KW: Investigation, Methodology, Writing – original draft, Writing – review & editing. JX: Investigation, Methodology, Writing – original draft, Writing – review & editing. FZ: Data curation, Project administration, Writing – original draft, Writing – review & editing. VS: Conceptualization, Investigation, Project administration, Writing – original draft, Writing – review & editing.
